# Characterization of Rongchang piglets after infection with type 2 porcine reproductive and respiratory syndrome virus strains differing in pathogenicity

**DOI:** 10.3389/fmicb.2023.1283039

**Published:** 2023-10-18

**Authors:** Wenli Zhang, Wenjie Ma, Yu Pan, Xinrong Wang, Mengjie Wang, He Zhang, Junxin Gao, Hongliang Zhang, Zhijun Tian, Changwen Li, Hongyan Chen, Changyou Xia, Yue Wang

**Affiliations:** ^1^State Key Laboratory for Animal Disease Control and Prevention, Heilongjiang Provincial Key Laboratory of Laboratory Animal and Comparative Medicine, Harbin Veterinary Research Institute, Chinese Academy of Agricultural Sciences, Harbin, China; ^2^College of Veterinary Medicine, Southwest University, Chongqing, China; ^3^National Center of Technology Innovation for Pigs, Chongqing, China

**Keywords:** Rongchang pig, PRRSV, HuN4, SD53-1603, immune responses

## Abstract

Porcine reproductive and respiratory syndrome virus (PRRSV) affects the production and health of pigs and causes severe economic losses to the swine industry worldwide. Different pig breeds have been reported to have different levels of susceptibility to PRRSV, and different PRRSV strains may also influence the infectivity and pathogenicity of the virus. In this study, the susceptibility of Rongchang pigs (a prominent local pig breed in China) to PRRSV infection was thoroughly investigated. Rongchang piglets were exposed to two PRRSV strains: HuN4 (highly pathogenic PRRSV) and SD53-1603 (moderately virulent NADC30-like PRRSV). We observed that Rongchang pigs infected with HuN4 displayed significant clinical manifestations, including fever, reduced body weight, and interstitial pneumonia lesions. Routine blood tests revealed that HuN4-infected pigs exhibited slightly decreased levels of red blood cells, hemoglobin, reticulocytes, and a notable increase in monocytes than control pigs. Additionally, the Rongchang pigs exhibiting severe clinical signs presented a higher neutrophil-to-lymphocyte ratio and a lower lymphocyte-to-monocyte ratio. In contrast, SD53-1603 infection did not cause considerable harm to Rongchang pigs, only resulting in slightly elevated leukocytes and lymphocytes. Furthermore, these two PRRSV strains elicited divergent cytokine responses, such that SD53-1603 infection induced higher levels of TNF-α and IFN-γ, whereas HuN4 infection upregulated IL-1β. These dissimilarities in clinical symptoms, pathological changes, viremia, cytokine expression, and routine blood indices between HuN4 and SD53-1603 infections are critical in understanding the mechanisms of PRRSV infection and developing rational prevention and control strategies against PRRSV.

## Introduction

Porcine reproductive and respiratory syndrome (PRRS), first reported in 1987, has been a serious concern to the global pig industry. PRRS virus (PRRSV), a single-stranded positive-strand RNA virus belonging to the *Arteriviridae* family, is the causative agent of PRRS. PRRSV is classified into two different species: Betaarterivirus suid 1 (PRRSV-1, formally European PRRSV) and Betaarterivirus suid 2 (PRRSV-2, formally North American PRRSV) according to significant antigenic variation, which share about 60% genomic identity (Brinton et al., 2021). Since PRRS was initially identified in China in 1996, the predominant PRRSV strains in China have been PRRSV-2. Notably, PRRSV exhibits high genetic diversity and evolutionary rates (Fang et al., 2022). The highly pathogenic PRRSV (HP-PRRSV) outbreak resulted in persistent high fever, severe organ damage or lesions, and high morbidity and mortality in central China in 2006 (Tian et al., 2007; Tong et al., 2007). This devastating outbreak rapidly spread across China and subsequently affected several Southeast Asian countries, including Vietnam and Laos, culminating in the most severe disease outbreak in the Asian pig industry (Lin et al., 2020). In 2015, a novel PRRSV (NADC30-like) that is genetically similar to the NADC30 strain isolated in the United States in 2008 emerged in China and quickly disseminated throughout the country. Although PRRSV NADC30-like strains are generally less virulent than HP-PRRSV strains, they still elicit clinical respiratory symptoms and moderate pathogenicity in commercial pig breeds (Zhou et al., 2015; Tian, 2017). Currently, both HP-PRRSV and NADC30-like strains are the predominantly transmitted strains within pig population in China (Liu et al., 2022).

Notably, different pig breeds have shown different levels of susceptibility to PRRSV infections *in vitro* and *in vivo* (Meng et al., 2018). For example, native pig breeds exhibited better resistance to HP-PRRSV strain than commercial breeds like the Large White pig and Landrace pig (Xing et al., 2014; Liang et al., 2016). Among the native breeds, the Rongchang pig, one of Chinese local breeds, serves as an exemplary model for biomedical research purposes (Gan et al., 2015; Chen et al., 2017). In this study, we thoroughly compared the clinical characteristics, viral loads, routine blood tests, and cytokine levels of Rongchang pigs infected with either HP-PRRSV strain HuN4 or NADC30-like PRRSV strain SD53-1603 to evaluate immune responses of Chinese local breed to different virulent PRRSV strains.

## Materials and methods

### Cells and viruses

The Marc-145 cell (an African green monkey kidney epithelial cell line, ATCC CRL-12231) was cultured in DMEM with 8% FBS and used for PRRSV propagation and titration. The HP-PRRSV strain HuN4 (GenBank accession number: EF635006.1) (PRRSV-2) was isolated from tested pig samples characterized by high fever and a high portion of deaths in China in 2006 (Tong et al., 2007). The NADC30-like PRRSV strain SD53-1603 (GenBank accession number: MH651744.1) was isolated from PRRSV-positive samples in China in 2016, which was less pathogenic than HP-PRRSV strains (Zhang et al., 2019).

### Animal experiments

Rongchang piglets were obtained from a historically PRRSV-negative farm free from anti-PRRS vaccines. Antigen and antibody tests were conducted for the presence of swine pathogens, including PRRSV, African swine fever virus (ASFV), classical swine fever virus (CSFV), pseudorabies virus (PRV), and porcine circovirus 2 (PCV2) by ELISA kits (INgzim, Spain) and normal PCR/RT-PCR. According to the constructed Random Table method (Sundaram et al., 2010), thirteen 4-week-old male Rongchang pigs free of PRRSV, ASFV, CSFV, PRV, and PCV2 were randomly assigned to three groups: the HuN4 infection group (*n* = 5), the SD53-1603 infection group (*n* = 5), and the control group (*n* = 3). The pigs in different groups were housed separately in biosafety rooms accordingly. Each piglet in the corresponding infection groups received intramuscular injections (2 mL) and nasal drips (2 mL) of either HuN4 or SD53-1603 (2.0 × 10^4^ TCID_50_/mL). Meanwhile, the control piglets received equal amounts of DMEM. Clinical signs and rectal temperatures of all experimental piglets were recorded daily, and body weights were measured at 7, 14, and 21 days post-infection (dpi). Blood samples were collected at 0, 2, 4, 7, 10, 14, 17, and 21 dpi. The piglets that were expected to die were euthanized and immediately necropsied, while the remaining piglets were euthanized and necropsied at 21 dpi. To quantify the presence or absence of PRRSV, tissue samples were collected from various organs, including heart, liver, spleen, lung, kidney, lymph node, tonsil, small intestine, bladder, and stomach. Among them, lung and lymph node tissues were fixed using 4% paraformaldehyde for histological examinations.

### Routine blood tests

Whole blood samples were collected using special anticoagulation tubes. Subsequently, blood samples were processed using the ProCyte Dx fully automated hematology analyzer (IDEXX, United States) to determine routine blood physiological indicators, including red blood cell count (RBC), hemoglobin concentration (HGB), platelet count (PLT), white blood cell count (WBC), percentage of monocytes (MONO), percentage of lymphocytes (LYMPH), percentage of neutrophils (NEUT), and others.

### Estimation of the viral load

Total RNA was extracted from blood and tissue samples using TRIzol following the manufacturer’s guidelines (Invitrogen, United States). The cDNA was synthesized by the reverse transcription kit containing oligo(dT) primer (TaKaRa, Japan) with 1 μg RNA per sample. The quantitative PCR (qPCR) was performed using the commercial TB Green® Fast qPCR Mix (TaKaRa, Japan) with specific primers listed in Table 1. The qPCR mixture consisted of 1 × TB Green Fast qPCR Mix, 0.2 μM each of the primers, cDNA (2 μL), and the volume was made up to 20 μL with deionized water. The qPCR conditions were as follows: denaturing at 95°C for 30 s, followed by 40 cycles at 95°C for 5 s and 60°C for 10 s and a melt curve from 72°C to 95°C. The Ct (cycle time) value of cDNA was determined by using a standard curve, which was generated using the known copy number of the recombinant plasmids that encompassed a conserved segment of the PRRSV ORF7 (Madapong et al., 2020).

**Table 1:** Primers used for the construction of reference plasmid and qPCR.

Primer name	Primer sequence (5′ – 3′)
PRRSV-ORF7-F	AGATCATCGCCCAACAAAAC
PRRSV-ORF7-R	GACACAATTGCCGCTCACTA

### Detection of cytokines and antibodies

Serum levels of IL-1β, IL-10, IFN-γ, TNF-α, and IgG were analyzed using corresponding ELISA kits following the manufacturer’s instructions (Cloud-Clone Corp, United States). Furthermore, serum samples were examined for PRRSV-specific antibodies using ELISA (IDEXX, USA), and a sample-to-positive ratio (S/P ratio) of ≥0.4 was considered positive. Multiple lung fractions were simultaneously taken, mixed and ground. The supernatants were then analyzed to determine antibody levels using ELISA (IDEXX, United States). Neutralizing antibody levels of pig sera at 21 dpi were performed on Marc-145 cells by using the corresponding virus strain for challenge as previously described (Ma et al., 2016).

### Statistical analysis

All data are presented as mean ± standard deviation (SD). Statistical analysis was performed using GraphPad Prism software (version 6.0) with two-tailed unpaired Student’s *t* tests to assess statistical differences between groups. Asterisks indicate statistical significance: NS, no significance; *, *p* < 0.05; **, *p* < 0.01; ***, *p* < 0.001.

### Animal welfare statement

Animal experiments were conducted strictly following the guidelines of the Animal Ethics Committee of Harbin Veterinary Research Institute, Chinese Academy of Agricultural Sciences, China. The approval number of the animal ethics committee is 200720–01.

## Results

### Clinical manifestations of two PRRSV strains in pigs

**Figure 1:**
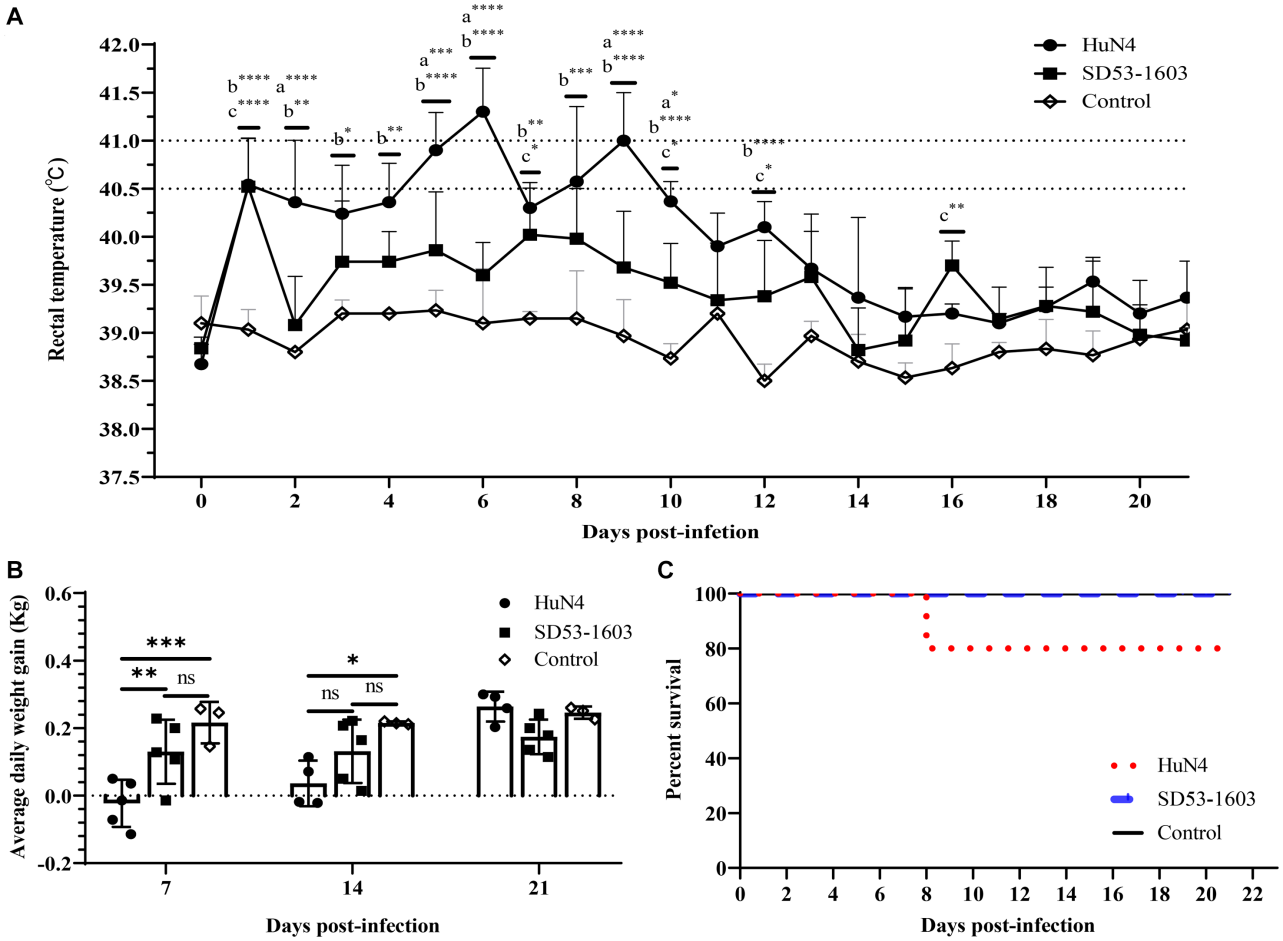
Temperature changes, average daily body weight, and survival rate in Rongchang piglets following PRRSV HuN4 or SD53-1603 or a control challenge. **(A)** Rectal temperatures of challenge and control group piglets. The fever cut-off value was set at 40.5°C. The mean temperatures ± standard deviation (SD) are presented. **(B)** Weight changes of piglets during the study period. All piglets were weighed at 7, 14, and 21 days post-infection (dpi). The average daily weight gain per group was calculated for days 0–7, 7–14, and 14–21 post-infection. The bars represent the average daily body weight of all surviving piglets ± standard deviation (SD). **(C)** Survival rate of Rongchang piglets inoculated with viruses or control. *, *p* < 0.05; **, *p* < 0.01; ***, *p* < 0.001; ****, *p* < 0.0001, a, HuN4 vs. SD53-1603; b, HuN4 vs. control; c, SD53-1603 vs. control.

During the whole study period, the control piglets did not exhibit any clinical signs related to PRRSV infection. HuN4-infected piglets displayed noticeable clinical symptoms, including labored breathing, transient anorexia, occasional episodes of diarrhea, and one pig succumbed to death at 8 dpi (Figure 1C). Moreover, HuN4-infected piglets experienced a high fever, with temperatures exceeding 40.5°C for a cumulative period of 5 days, peaking at about 41°C on two occasions (at 6 and 9 dpi) (Figure 1A). In contrast, SD53-1603-infected piglets exhibited only transient immobility and no other discernible clinical signs, and these piglets had no fever and all survived (Figures 1A, C). At 7 dpi, the average daily weight gain in the HuN4 infection group was significantly lower than the SD53-1603 infection and control groups. Even at 14 dpi, the weight gain of the HuN4 infection group remained lower than that of the SD53-1603 infection group; however, the disparity in average daily weight gain between the SD53-1603-infected and control piglets was not statistically significant over the course of the study (Figure 1B).

### Macroscopic and microscopic lesions in the lungs and lymph nodes

**Figure 2:**
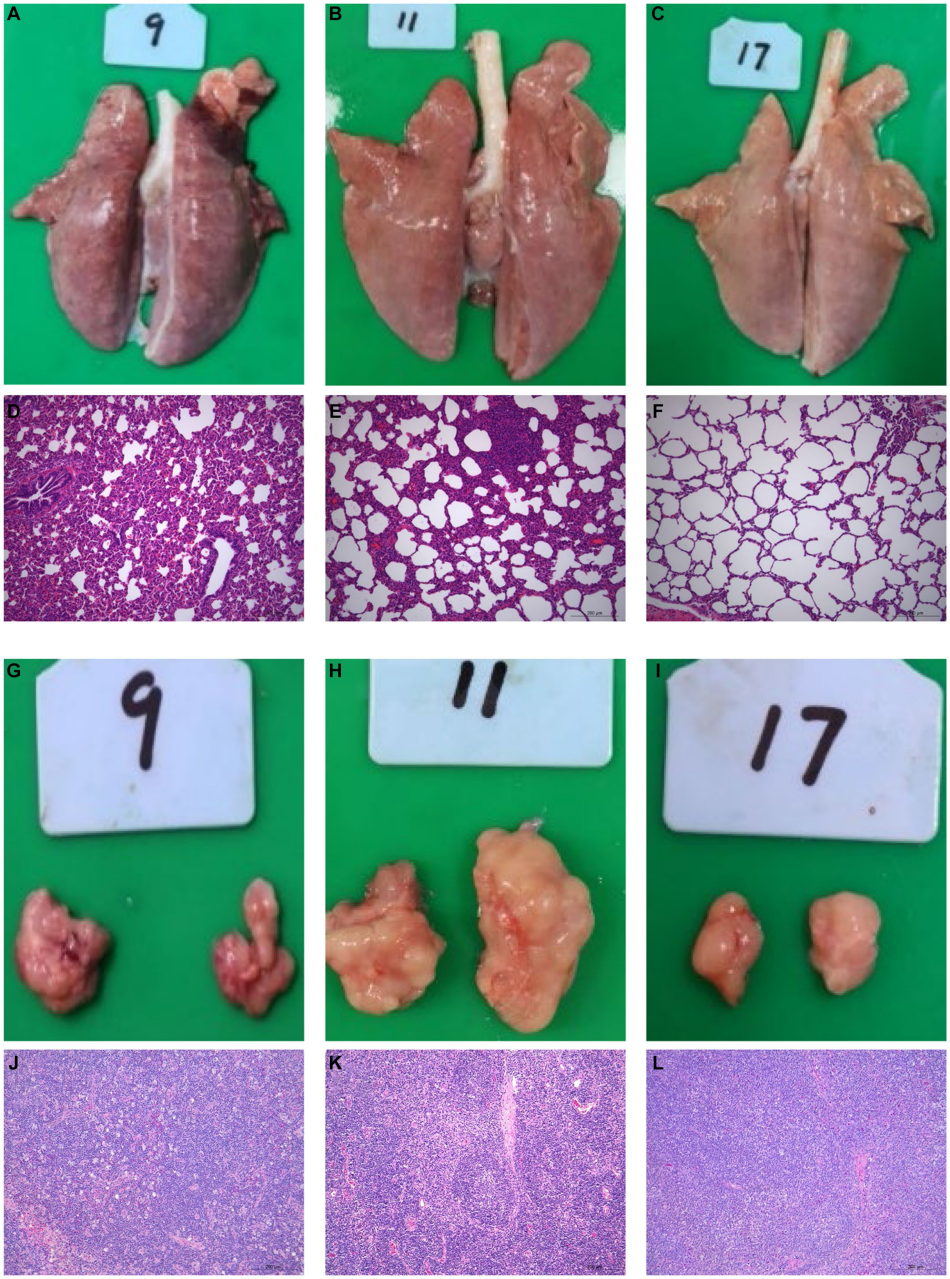
Gross and histological lesions of the lungs and lymph nodes of Rongchang piglets infected with HuN4 and SD53-1603 PRRSV strains. Lung tissues from piglets infected with HuN4 **(A,D)**, SD53-1603 **(B,E)**, and control piglets **(C,F)** were examined for anatomical and pathological changes. Additionally, submandibular lymph node tissues from piglets infected with HuN4 **(G,J)**, SD53-1603 **(H,K)**, and control piglets **(I,L)** were also evaluated. The original magnification of figures E, F, G, K, L, and M is 200 × .

All surviving pigs underwent dissection at 21 dpi for histopathological examinations. No histological abnormalities were observed in control piglets (Figures 2C, F, I, L). HuN4-infected piglets exhibited partial consolidation and hemorrhage in the lungs, along with inflammatory cell infiltration and alveolar epithelial cell hyperplasia, which resulted in moderate alveolar septal widening and a small amount of exudate in the alveolar lumen (Figures 2A, D). The submandibular lymph nodes showed hemorrhage, lymphocytopenia, and macrophage hyperplasia (Figures 2G, J). Variable degrees of swelling and hemorrhage were also observed in brain, liver, spleen, stomach, and intestinal segments. Furthermore, in HuN4-infected piglets, the spleen exhibited irregular edges, the thickness of the intestinal wall was reduced, and the thymus gland displayed atrophy to varying degrees (Supplementary Figure S1). On the contrary, SD53-1603-infected piglets did not show any marked damage to lungs (Figures 2B, E), lymph nodes (Figures 2H, K), or other organs (Supplementary Figure S1). These findings indicate that SD53-1603 induces less severe pathological changes than HuN4 in Rongchang piglets.

### Comparison of hematological responses

**Figure 3:**
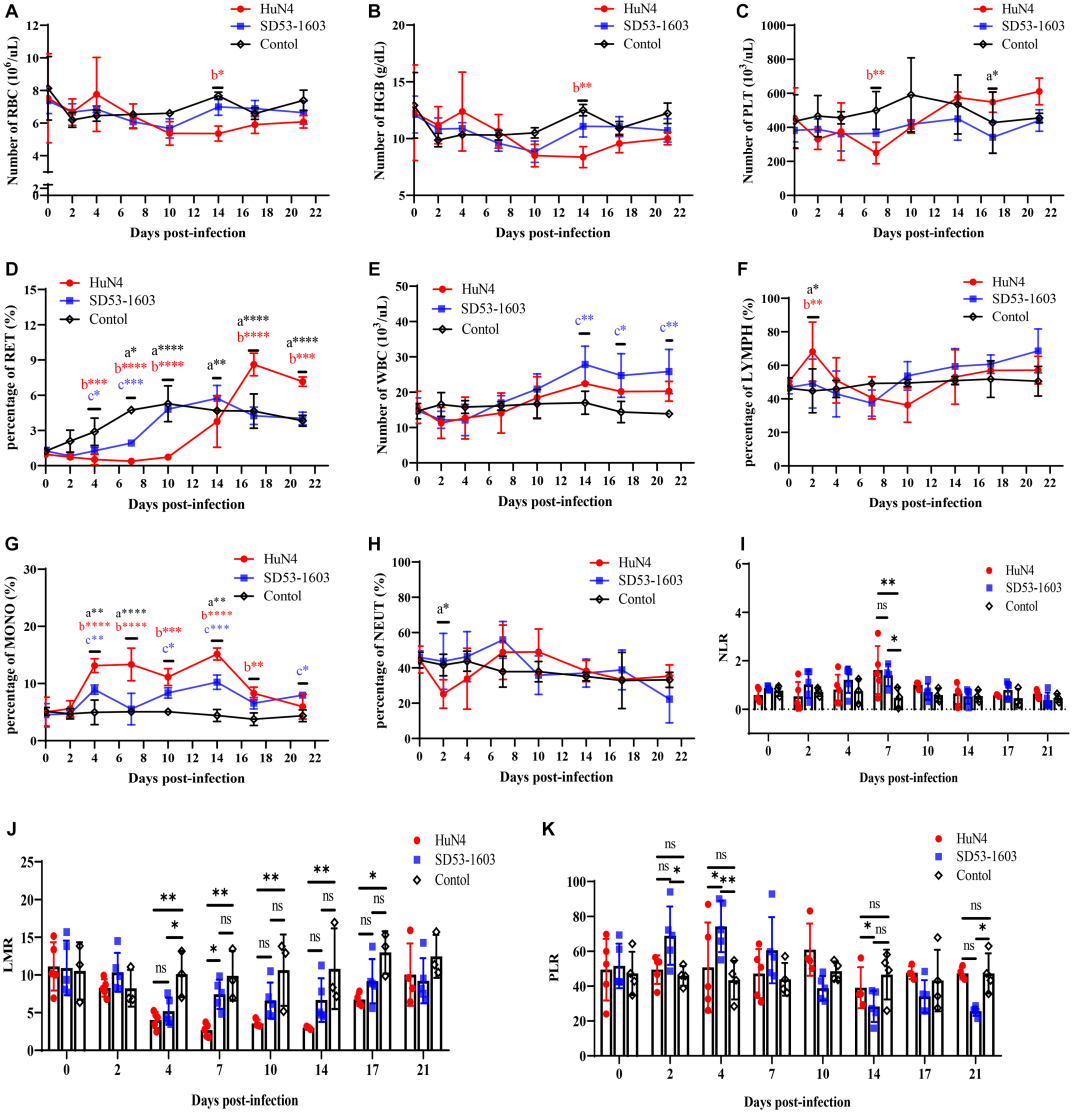
Routine blood analysis of piglets following PRRSV challenge. **(A)** Red blood cell count (RBC); **(B)** Hemoglobin concentration (HGB); **(C)** Platelet count (PLT). **(D)** Reticulocyte percentage (RET); **(E)** White blood cell count (WBC); **(F)** Lymphocyte percentage (LYMPH); **(G)** Monocyte percentage (MONO); **(H)** Neutrophil percentage (NEUT). **(I)** Neutrophil-to-lymphocyte ratio (NLR); **(J)** Lymphocyte-to-monocyte ratio (LMR); and **(K)** Platelet-to-lymphocyte ratio (PLR). *, *p* < 0.05; **, *p* < 0.01; ***, *p* < 0.001; ****, *p* < 0.0001, a, HuN4 vs. SD53-1603; b, HuN4 vs. control; c, SD53-1603 vs. control.

HuN4-infected piglets exhibited slightly lower levels of erythrocytes and hemoglobin compared to the control piglets at 10 dpi. At 7 dpi, the platelet count in HuN4-infected piglets was significantly lower than that of the control group. However, it gradually increased and ultimately reached slightly higher levels than the control piglets (Figures 3A–C). Furthermore, the reticulocyte counts were significantly lower in HuN4-infected piglets than in SD53-1603-infected and control piglets during the first 14 days, but increased to significantly higher levels from 14 to 21 dpi (Figure 3D). In contrast, the levels of erythrocytes, hemoglobin, platelets, and reticulocytes were similar between SD53-1603-infected and control piglets (Figures 3A–D). Compared to the HuN4 infection group, SD53-1603-infected piglets exhibited higher levels of leukocytes and lymphocytes at 7 dpi, but the differences between the means were not statistically significant (Figures 3E–F). However, HuN4-infected piglets had significantly higher levels of monocytes than the SD53-1603-infected piglets from 4 to 14 dpi, which then returned to the similar baseline level of the control group (Figure 3G). The neutrophil-to-lymphocyte ratio (NLR) was significantly higher in both infection groups compared to the control group at 7 dpi. Notably, at the time of death, the HuN4-infected piglet had an NLR of greater than 2 (Figure 3I). The lymphocyte-to-monocyte ratio (LMR) was decreased in both infection groups. HuN4-infected piglets exhibited significantly lower LMR than the control piglets at 4 dpi. Subsequently, the LMR achieved the lowest values at 7 dpi and eventually recovered to the matching level of the control group at 21 dpi. In contrast, SD53-1603-infected piglets exhibited a significant decrease in LMR only at 4 dpi, with no significant differences at the other time points compared to the control group (Figure 3J). Regarding the platelet-to-lymphocyte ratio (PLR), there were no significant changes in the HuN4 infection group. However, the PLR was higher in SD53-1603-infected piglets than the control piglets at 7 dpi and thereafter gradually decreased (Figure 3K).

### Viral RNA load in blood and tissues

**Figure 4:**
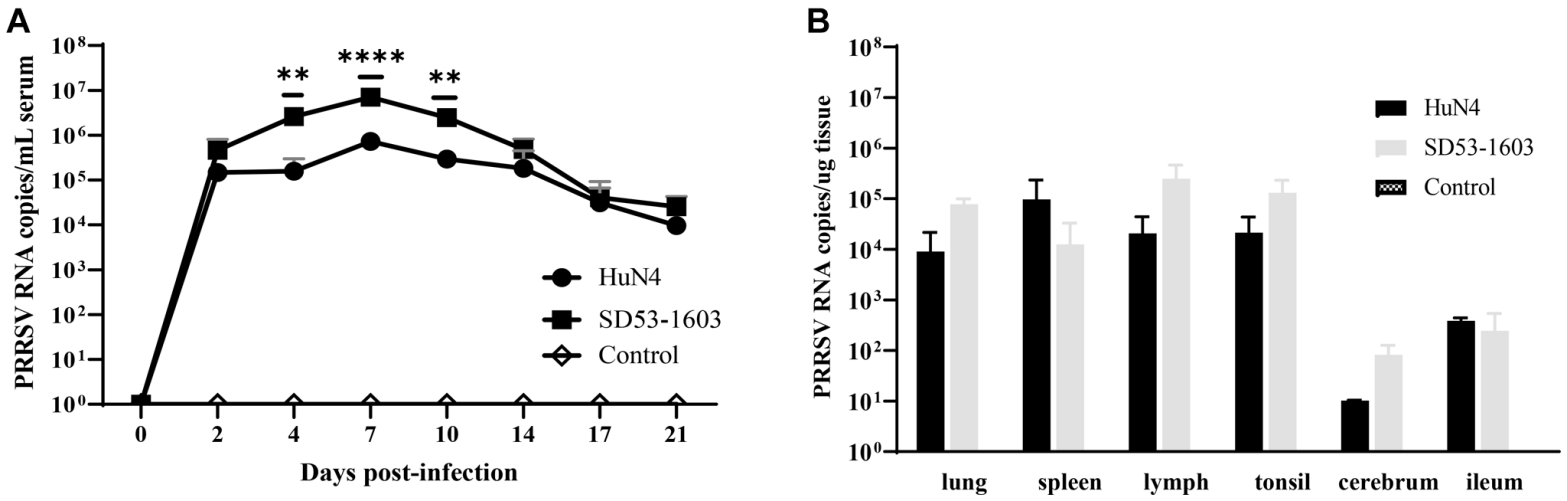
Viremia **(A)** and viral loads in six tissues **(B)** after HuN4 and SD53-1603 challenges. PRRSV viral RNA in sera and tissues was measured by qPCR. Each bar represents the group average of pigs ± SD **, *p* < 0.01; ****, *p* < 0.0001.

To investigate the disparities in viral load across tissues from two infection groups, we extracted total RNA from diverse tissues and performed real-time qPCR. The qPCR result showed that viral RNA load in serum from both infection groups displayed a prominent peak at 7 dpi. Nonetheless, an intriguing revelation emerged, as SD53-1603-infected piglets exhibited a significantly higher viral load in comparison to HuN4-infected piglets at 4–10 dpi. However, by 17 dpi, the viral RNA copies in the sera of both groups essentially converged (Figure 4A). Intriguingly, an in-depth examination of the viral load in respective organs revealed a remarkable disparity. Specifically, SD53-1603-infected piglets consistently exhibited higher levels of viral loads than HuN4-infected piglets, except in the spleen and ileum, although this difference was not statistically significant (Figure 4B). Additionally, viral RNA was detected in heart, liver, kidney, stomach, and brain of two infection groups (data not shown), which offers convincing evidence of the widespread histotropism of different pathogenic PRRSV strains in Rongchang pigs.

### Antibody responses in HuN4 and SD53-1603 infected piglets

**Figure 5:**
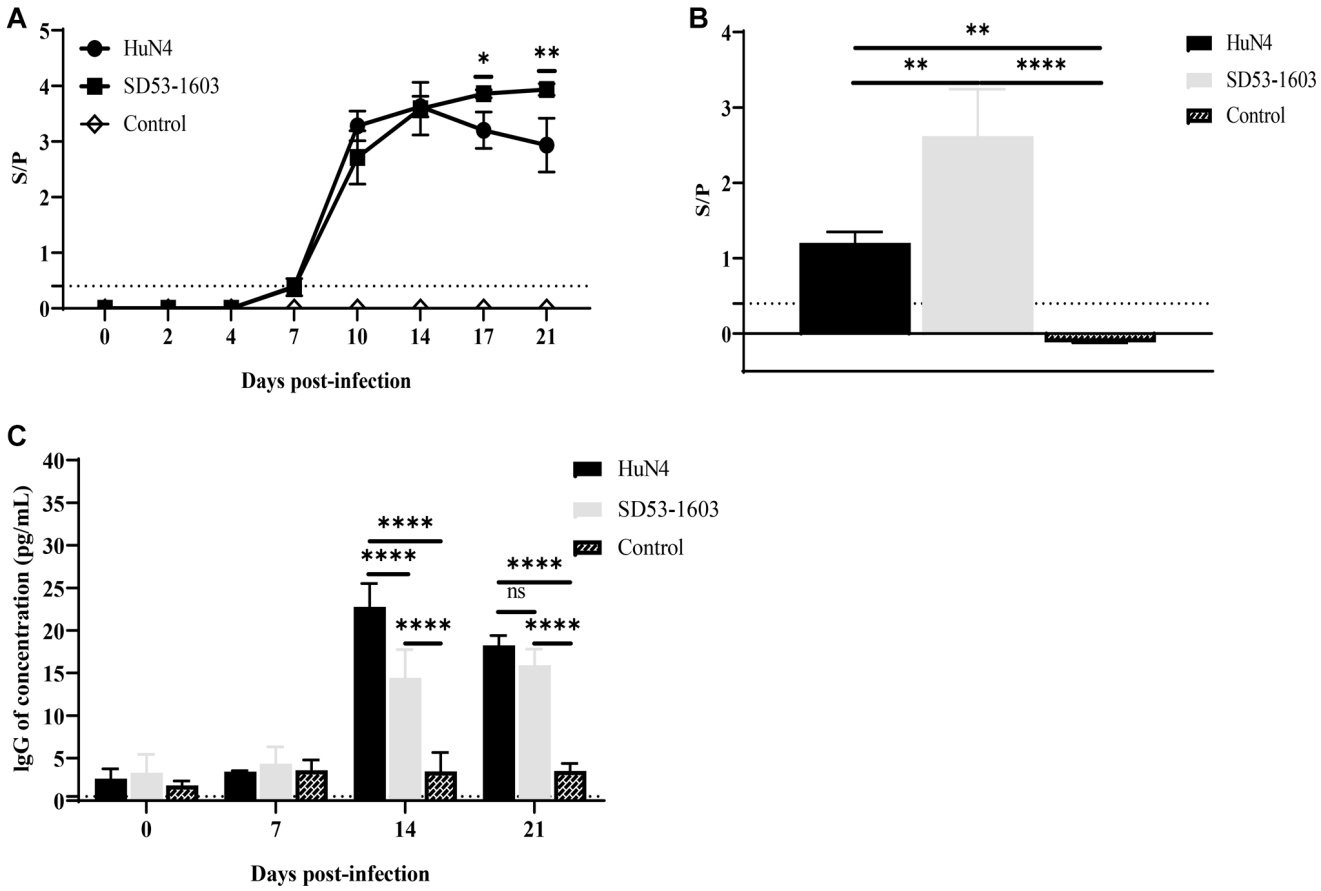
Antibody levels of Rongchang piglets following inoculation with HuN4, SD53-1603, or mock control. The levels of PRRSV-specific antibodies in lung **(A)** and serum **(B)**, and the levels of total IgG in serum **(C)** were determined using ELISA. The seroconversion threshold for PRRSV-specific antibodies was set at a sample-to-positive (s/p) ratio of 0.4. Each bar represents the mean ± SD.*, *p* < 0.05; **, *p* < 0.01; ****, *p* < 0.0001.

ELISA was conducted to examine the levels of PRRSV-specific antibodies and total IgG in the serum of infected piglets. Notably, we observed a rapid development of antibodies in the blood serum of both HuN4 and SD53-1603-infected piglets at 7 dpi. The antibody levels in HuN4-infected piglets peaked after 14 dpi, and then gradually declined. In contrast, SD53-1603-infected piglets displayed slightly lower antibody levels than HuN4-infected piglets at 10 dpi. However, between 17 and 21 dpi, the antibody levels in SD53-1603-infected piglets gradually increased and were significantly higher than those in HuN4-infected piglets (Figure 5A). The results of virus neutralization test showed that neither HuN4 nor SD53-1603 induced the detectable levels of effective neutralizing antibodies at 21 dpi (data not shown). Furthermore, the levels of PRRSV-specific antibodies in pulmonary abrasive fluid were significantly higher in SD53-1603-infected piglets than in HuN4-infected and control piglets at 21 dpi (Figure 5B). Total IgG levels in serum of the control piglets remained constant throughout the experimental phase. In contrast, there was no significant changes in IgG serum levels in either of the two infection groups during the initial 7 days. Interestingly, between 14 and 21 dpi, the IgG levels in both infection groups reached a harmonious equilibrium. However, at 14 dpi, HuN4-infected piglets had significantly higher IgG levels than SD53-1603-infected piglets (Figure 5C).

### Expression levels of cytokines in serum

**Figure 6:**
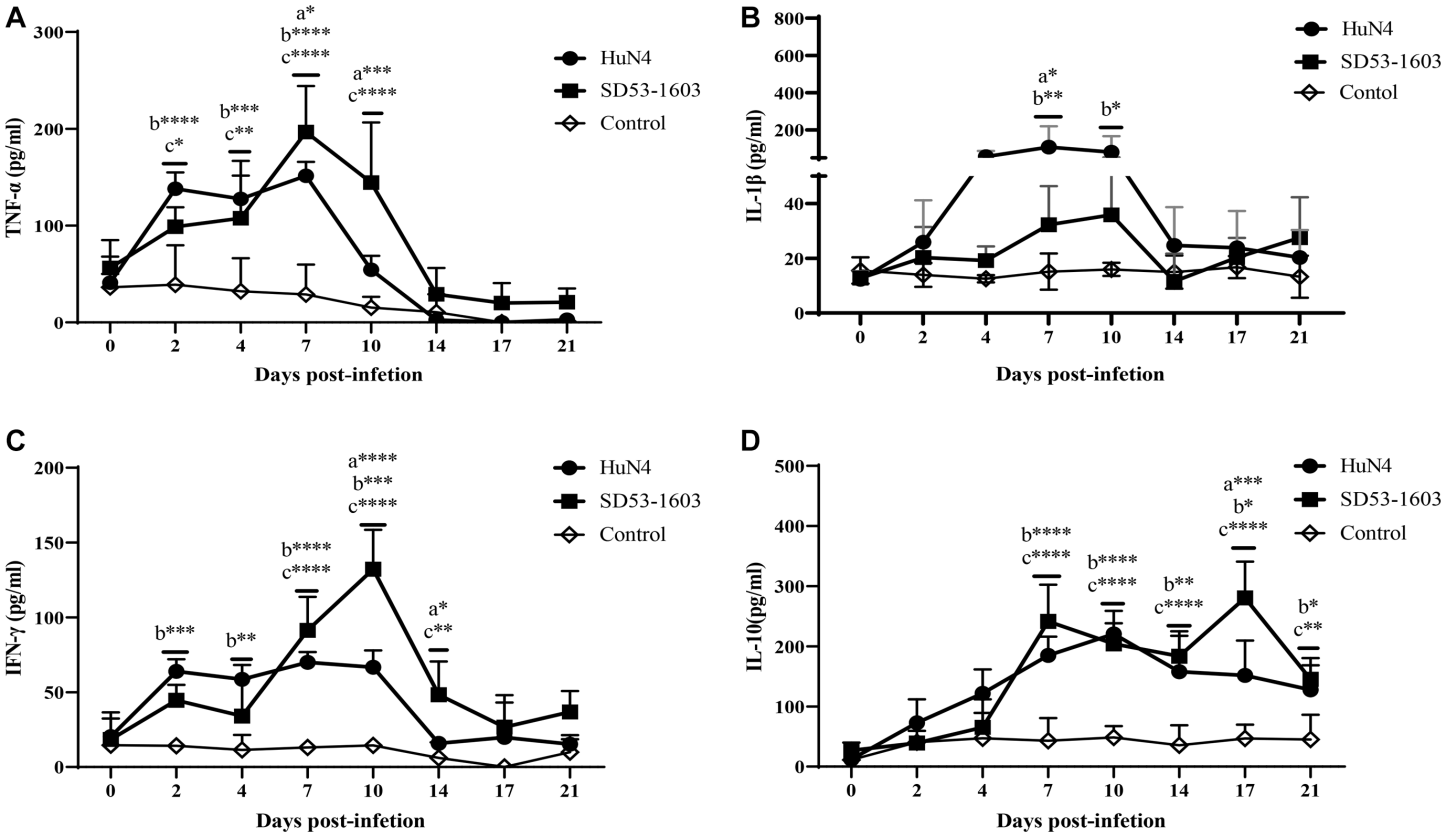
Cytokine levels in serum of HuN4 and SD53-1603 infected and control Rongchang piglets. The levels of TNF-α **(A)**, IL-1β **(B)**, IFN-γ **(C)**, and IL-10 **(D)** were measured at 0, 2, 4, 7, 10, 14, 17, and 21 dpi. *, *p* < 0.05; **, *p* < 0.01; ***, *p* < 0.001; ****, *p* < 0.0001, a, HuN4 vs SD53-1603; b, HuN4 vs control; c, SD53-1603 vs control.

The expression levels of different cytokines in serum were determined by ELISA. The results showed that cytokine profiles after HuN4 and SD53-1603 infection exhibited similar trends, but the extent of change differed between the two infection groups. The levels of TNF-α, IL-1β, and IFN-γ were first elevated after infection and then returned to baseline at 14 dpi after the peak (Figures 6A–C). During the first 4 days, HuN4-infected piglets showed slightly higher levels of TNF-α, IL-1β, IFN-γ, and IL-10 compared to SD53-1603-infected piglets. From 7 to 14 dpi, the HuN4 infection group exhibited higher levels of IL-1β, whereas the SD53-1603 infection group displayed higher levels of TNF-α and IFN-γ. The IL-10 levels gradually increased after infection with two strains within the first 10 days, and at 17 dpi SD53-1603-infected piglets had significantly higher levels of IL-10 than HuN4-infected piglets (Figure 6D).

## Discussion

Since 1996, multiple PRRSV subtypes have widely spread in China, causing significant economic losses. Recently, HP-PRRSV-like and NADC30-like strains have been the major circulating strains in the fields and result in devastating destruction to swine industry (Zhou et al., 2022; Li et al., 2023; Long et al., 2023). In this study, we used Rongchang piglets (a local Chinese breed) to determine the pathogenicity of two different PRRSV strains, HP-PRRSV strain HuN4 and NADC30-like strain SD53-1603. In general, Rongchang piglets infected with HuN4 generally experience more severe clinical syndromes and higher mortality rates than those infected with SD53-1603. More specifically, Rongchang piglets infected with HuN4 exhibited a short period of hyperthermia (≥41°C), with 20 % of the pigs succumbing to the virus infection. However, the surviving piglets remained healthy both clinically and anatomically (Supplementary Figure S2). In comparison, Rongchang pigs infected with SD53-1603 exhibited minimal respiratory tract, along with other related clinical symptoms and classic pathological changes. The average temperature of Rongchang piglets infected with SD53-1603 was below 40.5°C, and all pigs survived. Previous reports have demonstrated that different PRRSV strains lead to variable clinical syndromes, and different pig breeds have different susceptibility to PRRSV infection (Xing et al., 2014; Liang et al., 2016; Meng et al., 2018; You et al., 2023). Two Chinese pig breeds, such as Tongcheng pigs and Dapulian pigs, have showed greater resistance to HP-PRRSV infection than Large White pigs (Xing et al., 2014; Liang et al., 2016). Based on clinical symptoms, pathological changes, mortality rates, and the status of surviving piglets reported in previous studies of HuN4 (Zhou et al., 2008; Tian et al., 2009; Wang et al., 2018) and SD53-1603 (Zhang et al., 2019) infections in Large White piglets, we infer that Rongchang piglets may exhibit a greater resistance to PRRSV compared to Large White piglets. However, further research is required to substantiate this conclusion.

The levels of viral load are associated with disease severity and mortality and can predict disease outcomes. However, in the case of Rongchang pigs infected with HuN4 or SD53-1603, the viral pathogenicity was not only related to the viral load in serum and lung. Despite the higher pathogenicity of HuN4, it replicated at relatively lower levels compared to SD53-1603 in Rongchang pigs. Additionally, the levels of PRRSV-specific antibodies in serum and lung indicated that SD53-1603 infection produced higher levels of N protein-specific antibodies than HuN4 infection. These data suggest that SD53-1603 replicates more efficiently than HuN4 in Rongchang pigs in serum. Higher viral loads have also been reported in serum from infected pigs with certain low pathogenicity (Morgan et al., 2013; Zhang et al., 2013; Ma et al., 2022). Previous data have demonstrated that viral nsp2, nsp9 and nsp10 play critical roles in determining viral replication efficiency and fatal virulence of HP-PRRSV (Li et al., 2014; Kong et al., 2023). It is worth mentioning that the nsp2 of HP-PRRSV strain plays an important role in viral clearance in lung and lymphoid tissues, which may explain the lower levels of HP-PRRSV HuN4 load than the moderately virulent strain SD53-1603 in Rongchang pigs. Additionally, the magnitude of viral load can be influenced by various factors, including antibody levels, receptor expression levels, transmission efficiency, and others (Frydas et al., 2013; Morgan et al., 2013; Rodriguez-Gomez et al., 2019).

In this study, we observed the absence of neutralizing antibodies in the serum of Rongchang pigs infected with both virus strains at 21 days, which suggests a delay in the production of neutralizing antibodies in these pigs. IgG is primarily responsible for limiting viremia and systemic transmission of PRRSV. The elevated levels of IgG antibodies in Rongchang pigs infected with HuN4 indicate that compared to SD53 infection, HuN4 infection poses a more severe threat to the immune system, resulting in a broader and stronger antibody response. High levels of IgG antibodies are also commonly observed in severely ill patients following COVID-19 infection (Zhang et al., 2020). Antibody response may be associated with organ damage in addition to antiviral efficacy (Tantuoyir and Rezaei, 2021). The IgG levels in the serum can be used to generate a combined immune response phenotype to predict the course of disease progression (Zhang et al., 2020).

Routine blood tests are widely used in clinical practice for the timely detection of physiological and pathological changes in animals. HuN4-infected Rongchang pigs exhibited lower levels of reticulocytes, erythrocytes, and hemoglobin compared to the control group, indicating a potential inhibition of hematopoietic function in pigs. Additionally, the platelet levels were significantly lower in HuN4-infected Rongchang piglets than in control piglets at 7 dpi. This indicates that HuN4 infection-induced thrombocytopenia may be associated with abnormal coagulation function. Meanwhile, HuN4 infection significantly upregulated monocyte levels, which may play a crucial role in controlling viremia but may also induce tissue damage during infection (Zhang et al., 2020). Moreover, Zika virus infection has been shown to enhance monocyte migration and virus propagation to neuronal cells by manipulating monocyte adhesion properties (Ayala-Nunez et al., 2019), which may be an important mechanism for viral tissue invasion and persistence. On the contrary, SD53-1603 infection did not significantly affect the erythroid lineage and monocytes but resulted in increased levels of leukocytes and lymphocytes. NLR, LMR, and PLR are essential inflammation markers that can help predict infection severity (Kosidło et al., 2023). NLR values increased in both groups after infection, but the values were slightly higher in the SD53-1603 infection group than the HuN4 infection group during the first 7 days. Furthermore, an NLR value of greater than 2 was observed in the HuN4-infected piglet that died at 8 dpi. This suggests significantly higher NLR value may indicate a more severe disease (Damar Cakirca et al., 2021). The LMR decreased in both infection groups, and the HuN4 infection group had significantly lower LMR values than the control group after 4 dpi, while the SD53-1603 infection group did not show significant differences compared to the control group. In COVID-19 patients, more severe cases exhibited mononucleosis, resulting in decreased LMR (Kosidło et al., 2023), and the value decreased with disease severity (Citu et al., 2022). Although the PLR is considered a highly valuable non-specific marker of inflammation (Gujar et al., 2021), the PLR values in Rongchang pigs infected with two distinct PRRSV strains are not correlate with the associated symptoms. Additional data are needed to confirm whether the PLR can be used as an indicator for systemic inflammation following PRRSV infection in piglets.

Cytokines, including IL-1β, IL-10, IFN-γ, and TNF-α, play a critical defense role against PRRSV. IL-1β is a pro-inflammatory cytokine produced by immune cells such as macrophages and monocytes in response to infections or injuries, which can regulate immune responses, including fever, inflammation, and activation of other immune cells. The relatively high levels of IL-1β in Rongchang pigs from 2 to 10 days after HuN4 infection might contribute to the persistent increase in body temperature from 2 to 12 dpi and acute lung injury in HuN4-infected Rongchang pigs (Amarilla et al., 2015; Liang et al., 2016). TNF-α, another pro-inflammatory cytokine mainly produced by macrophages and T cells, plays a crucial role in inflammation and the immune response. Compared with the pigs inoculated with low pathogenic PRRSV, HP-PRRSV-infected Large White pigs induces higher levels of TNF-α (Zhang et al., 2013; Han et al., 2014). However, TNF-α levels were significantly increased in SD53-1603-infected Rongchang pigs at 7 dpi compared to HuN4-infected pigs. Previous studies have shown that Nsp1β and Nsp11 are responsible for HP-PRRSV-induced suppression of TNF-α production, leading to TNF-α production differences in PAM (He et al., 2015). IFN-γ participates in regulating the activity of immune cells, enhancing resistance to viruses by directly inhibiting virus replication and transmission. After 7 to 14 days of SD53-1603 infection, Rongchang pigs exhibited higher levels of IFN-γ, which may be associated with the larger viral load of SD53-1603. The balance between IL-10 and IFN-γ plays a crucial role in immune response (Sassi et al., 1999; Diaz et al., 2006; Karakhanova et al., 2014; Marcal et al., 2020). The IL-10 levels in Rongchang pigs remained relatively constant after infection with two PRRSV strains of different pathogenicity. However, SD53-1603-infected piglets showed significantly higher levels of IL-10 than HuN4-infected piglets at 17 dpi. IL-10 is generally considered an anti-inflammatory factor with a suppressive effect on immune response (Moore et al., 2001). It seems that the immune system of SD53-1603-infected Rongchang pigs attempts to balance the inflammatory response and return to a normal state. Briefly, immune responses induced by HuN4 and SD53-1603 infections shared some similarities, such as the elevation and recovery of IL-1β, TNF-α, and IFN-γ levels. However, they exhibited variations both in terms of timing and extent. These differences may reflect variations in the mode and mechanism of immune response after PRRSV infection.

Altogether, this is the first report to investigate two different PRRSV strains (HP-PRRSV strain HuN4 and NADC30-like strain SD53-1603) infection in Rongchang piglets. Our findings indicate that HuN4 is significantly more pathogenic than SD53-1603 in Rongchang pigs. Interestingly, the higher virus loads of SD53-1603 in animals might explain their recent widespread circulation in pig herds in China. Further research is needed to understand the molecular mechanisms of Rongchang piglets’ defense against PRRSV infection.

## Data availability statement

The original contributions presented in the study are included in the article/Supplementary material, further inquiries can be directed to the corresponding authors.

## Ethics statement

The animal study was approved by Animal Ethics Committee of Harbin Veterinary Research Institute, Chinese Academy of Agricultural Sciences, China. The approval number of the animal ethics committee is 200720-01. The study was conducted in accordance with the local legislation and institutional requirements.

## Author contributions

WZ: Conceptualization, Data curation, Formal analysis, Investigation, Methodology, Software, Validation, Visualization, Writing – original draft, Writing – review & editing. WM: Formal analysis, Software, Validation, Visualization, Writing – review & editing. YP: Software, Visualization, Writing – review & editing, Formal analysis. XW: Software, Visualization, Writing – review & editing, Formal analysis. MW: Software, Writing – review & editing, Formal analysis. HeZ: Software, Writing – review & editing. JG: Writing – review & editing, Formal analysis. HoZ: Writing – review & editing, Resources. ZT: Resources, Writing – review & editing. CL: Resources, Writing – review & editing. HC: Funding acquisition, Writing – review & editing. CX: Resources, Writing – review & editing. YW: Formal analysis, Funding acquisition, Methodology, Project administration, Resources, Software, Supervision, Validation, Visualization, Writing – original draft, Writing – review & editing.
