# Supplementary figures and images for: Characterization of Rongchang piglets after infection with type 2 porcine reproductive and respiratory syndrome virus strains differing in pathogenicity
Source: Front Microbiol. 2023 Oct 18;14:1283039. doi: 10.3389/fmicb.2023.1283039 (PMC10618352; doi:10.3389/fmicb.2023.1283039)

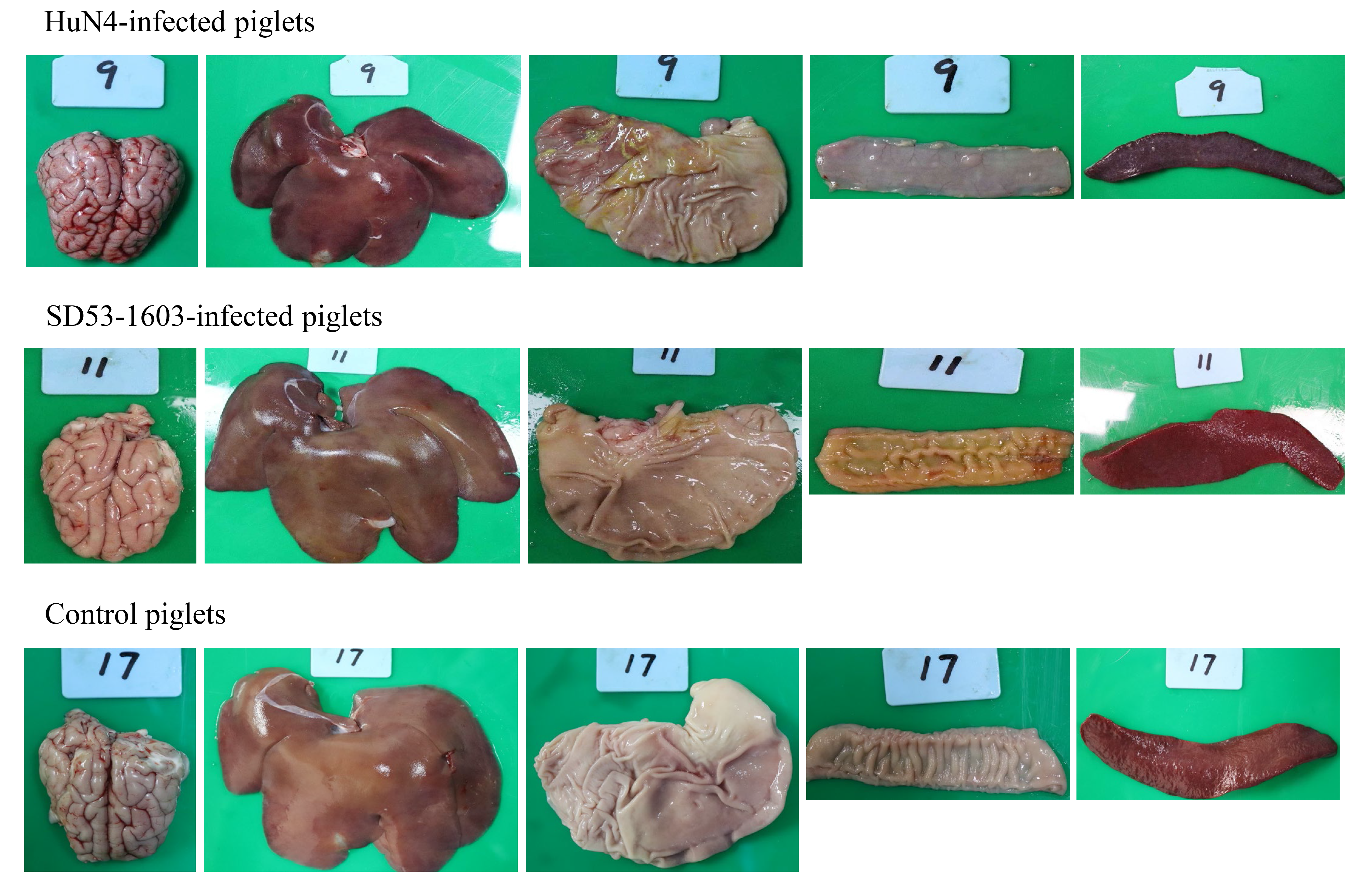

Supplement: Supplementary file 1 [file Image_1.TIF]

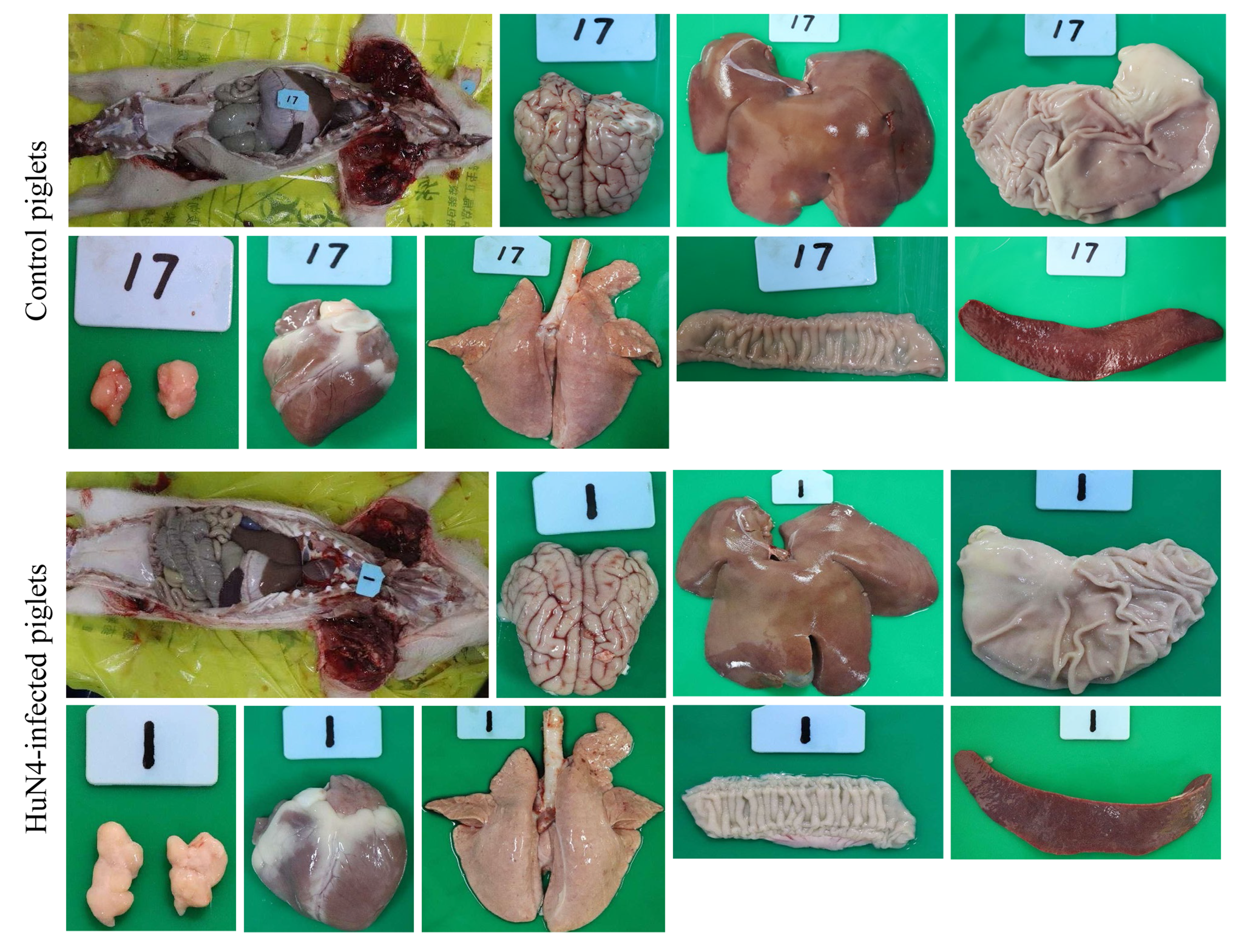

Supplement: Supplementary file 2 [file Image_2.TIF]
